# Antibiotics-induced intestinal dysbacteriosis caused behavioral alternations and neuronal activation in different brain regions in mice

**DOI:** 10.1186/s13041-021-00759-w

**Published:** 2021-03-06

**Authors:** Pan Wang, Ke Tu, Peng Cao, Yuefan Yang, Hao Zhang, Xin-Tong Qiu, Ming-Ming Zhang, Xiao-Jun Wu, Hui Yang, Tao Chen

**Affiliations:** 1 Institute of Medical Research, Northwestern Polytechnical University, Xi`an, Shaanxi 710072 P. R. China; 2 Department of Human Anatomy, Histology and Embryology & K.K. Leung Brain Research Centre, The Air Force Medical University, No. 169 Changle West Road, Xi’an, 710032 China; 3 Department of Anesthesiology, General Hospital of Tibet Military District, Lhasa, Tibet 850007 P. R. China; 4 Department of Biomedical Engineering, The Air Force Medical University, Xi`an, China; 5 Key Laboratory for Space Bioscience and Biotechnology, School of Life Sciences, Northwestern Polytechnical University, Xi`an, China; 6 Department of Neurosurgery, Fudan University Shanghai Cancer Center, 270 Dongan Road, Xuhui, 200032 Shanghai China

**Keywords:** Intestinal dysbacteriosis, Behavior, Antibiotics, Probiotics, Mice

## Abstract

Antibiotics affect gut microbial composition, leading to Gut–Brain-Axis imbalance and neurobehavioral changes. However, the intestinal dysbacteriosis associated behavior changes are not consistently reported. It is not clear whether these changes are transient or permanent. The neuroprotective effect of probiotics against intestinal dysbacteriosis induced alternations needs to be determined either. In the present study, oral antibiotic mixture including Ampicillin, Streptomycin, and Clindamycin was utilized to induce intestinal dysbacteriosis in mice. Antibiotics application triggered mechanical allodynia in *von frey* test and spontaneous pain in open field test. It also resulted in increased anxiety and depressive-like behaviors and damaged spatial memory performance. After application of probiotics, the mechanical allodynia and spontaneous pain were alleviated significantly. The anxiety behaviors, depressive-like behaviors and recognitive performance were ameliorative as well. By using Fos protein as a marker, it is found that the sensory, emotion and memory related brain regions were activated in mice with intestinal dysbacteriosis. Our study is not only helpful for enriching our basic knowledge for understanding the changed pain responses and related brain disorders in antibiotics-induced dysbacteriosis mice, but also beneficial for providing a more comprehensive mechanistic explanation for the regulation of antibiotics and probiotics on gut microbiota and relevant alternations in animal neurological behaviors.

## Introduction

Gut microbiota comprised a complex community of microbes, is widely reported to affect multiple physiological processes of the host, including gut-brain communications, development and function of brain, and behaviors [1]. The clinical manifestation inflammatory conditions and irritable bowel syndrome (IBS) are clear examples proving intestinal dysbacteriosis is closely connected with different pathological states of nervous system [2, 3]. However, the gut microbiota induced neuropathic condition is yet controversial. Recent studies indicate that antibiotic application in young healthy rodents induces depressive behaviors but not changing pain responses in the hot plate test [4]. While another study reports that oral application of antibiotics attenuates mechanical allodynia and thermal hyperalgesia in chronic-constriction injury of the sciatic nerve model mice [5]. However, it is highly likely that the pain responses should be deteriorated in animals with intestinal dysbacteriosis, since several epidemiological studies and our previous study indicated that spontaneous pain and emotional changes can be frequently observed in IBS patients and IBS mice [6, 7].

Besides, it is far from clear whether probiotic treatment will rescue the microbiota induced brain disorders. It is illustrated that Gut-Brain-Axis imbalance, due to intestinal dysbateriosis induced by oral ampicillin, leads to reduced cognition ability and decreased hippocampal neuronal density, which is partially reversed by probiotic treatment [8]. While another study points out a completely different conclusion that disturbances to gut microbiome by antibiotics has no impact on cognitive behaviors in rats [9]. Moreover, although probiotics have been proposed as a potential remedy for antibiotic-induced dysbacteriosis, the efficacy and underlying mechanisms remains unanswered so far. Thus, further exploration should be conducted to identify the inconsistent results regarding the pain responses and related brain disorders. Pain is an integrated symptom with sensory-discriminative, affective-motivational, and cognitive-evaluative changes [10]. It is important to examine the sensory dysfunction of nociceptive information (hyperalgesia, allodynia, and spontaneous pain), as well as various pain related brain disorders such as depression, anxiety and amnesia in animals with intestinal microflora imbalance.

In the present study, we used an intestinal dysbacteriosis model by exposing healthy mice to a broad-spectrum cocktail mixture of Ampicillin, Streptomycin and Clindamycin (ASC antibiotics) for 3 weeks [4, 11]. It’s shown that intestinal dysbacteriosis induced nociceptive responses, anxiety- and depression-like behaviors, and damaged spatial memory ability, as well as changed Fos expressions in cortex and hippocampus. The present results thus show a link among intestinal bacteriosis, behavior changes and altered neuronal excitability in certain brain regions. Furthermore, these changes were partially rescued by antibiotics withdrawal and/or probiotics application. This study aims to provide direct evidence for the changed neurological behaviors and neuronal activities in mice with intestinal dysbacteriosis and improving our understanding for the correlation in Gut-Brain-Axis.

## Results

### Application of antibiotic induced intestinal dysbacteriosis in mice

**Fig. 1:**
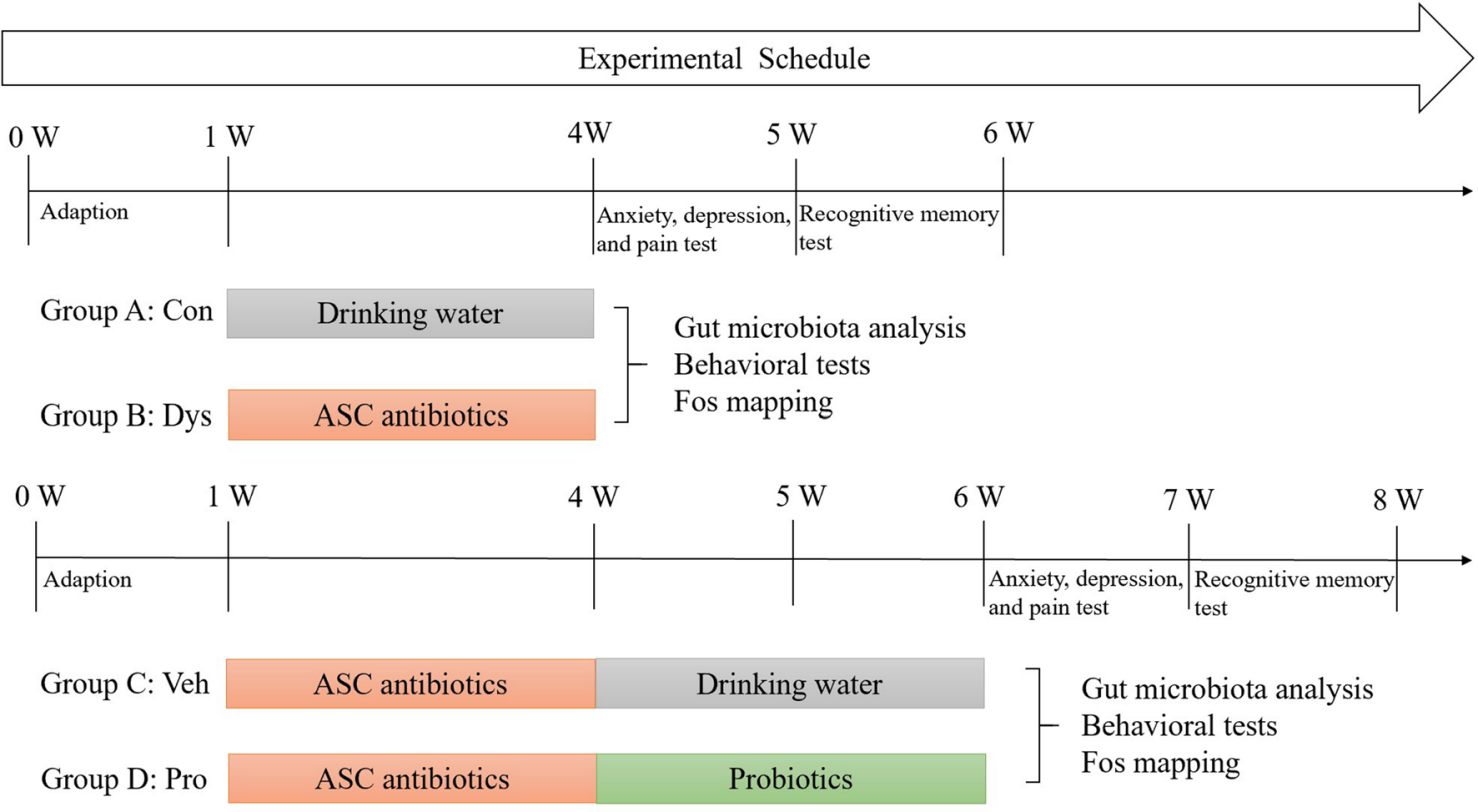
Experimental groups and schedule. Mice of group A (Control, Con) were treated with drinking water for 3 weeks, and mice of group B (Dysbacteriosis, Dys) were treated with 1 mg/ml ASC antibiotics (a mixture of Ampicillin, Streptomycin, and Clindamycin) for 3 weeks. While in Group C (Vehicle, Veh) and D (probiotics, Pro), there was a reverse course with ASC antibiotic mixture changed to drinking water and probiotics for another 2-week. The mice in each group were then subjected to gut microbiota analysis, a series of behavioral tests including anxiety, depression, pain test and recognitive memory test and immunostaining for Fos protein

The experimental timelines of each group were shown in Fig. 1. After habituation, mice were allocated to four groups and subjected to 3-week drinking solution processing—plain water for Group A (Con), and ASC antibiotic cocktail mixture for Group B (Dys), C (Veh) and D (Pro). While in Group C and D, there was a reverse course with ASC antibiotic mixture changed to plain water or probiotics for another 2 weeks. The mice in each group were then subjected to gut microbiota analysis, a series of behavioral tests including anxiety, depression, pain response test and recognitive memory test and immunostaining for Fos protein.

**Fig. 2:**
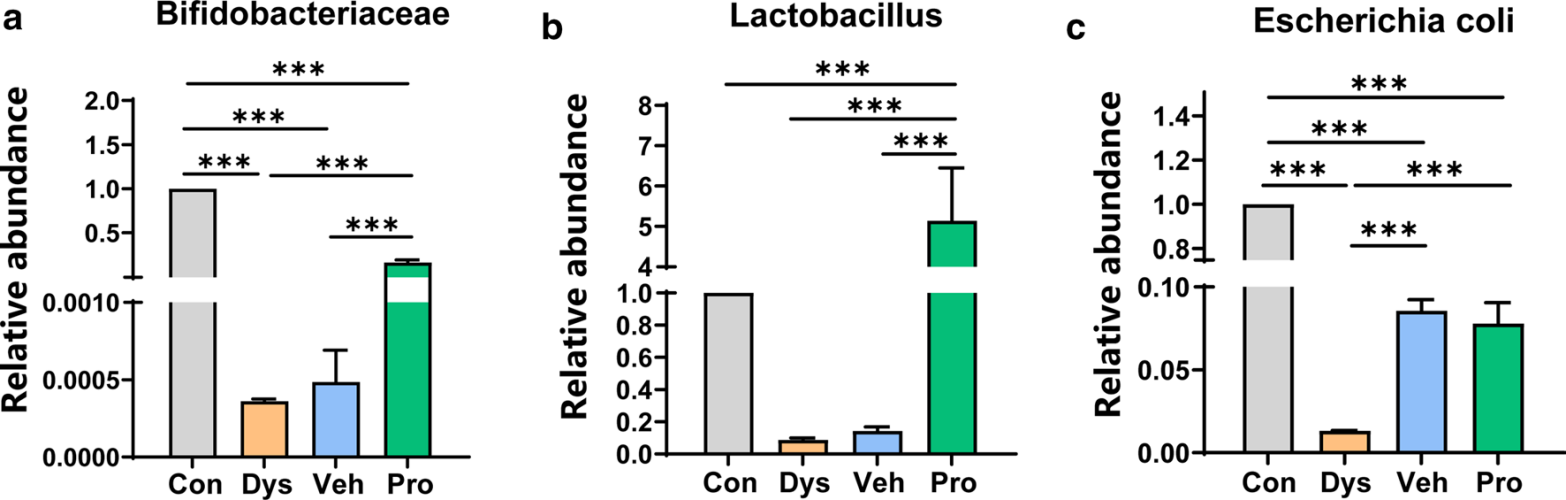
Effects of vehicle and probiotics on the abundance of gut bacteria in antibiotic-treated mice. The alternation of three main bacterial species was assessed with genus-specific primers targeting the 16S rRNA genes of **a** Bifidobacterium, **b** Escherichia Coli and **(C)** Lactobacillus. To better visualize the change within each genus, the relative abundance of each group has been divided by the mean of relative abundance obtained in the Con group, which corresponds to the value 1. Results are presented as means ± s.e.m., 8 pieces of pellet were collected per group, n = 6 mice per group (one-way ANOVA). ***p < 0.001

To investigate the possible changes in the gut microbiota composition, qPCR was performed to assess the alternation of three main bacterial species with genus-specific primers targeting the 16S rRNA genes of Bifidobacterium, Escherichia Coli, and Lactobacillus (Table 1). As expected, dysbacteriosis was induced in Dys mice treated with ASC antibiotics, in which the relative expression levels of three bacterial groups was reduced significantly when compared with the Con mice (Fig. 2). With the replacement of ASC mixture with probiotics but not plain water, the population of Bifidobacteriaceae and Lactobacillus were both recovered. However, the population of Escherichia coli was partially but significantly recovered, when the ASC mixture was replaced with either plain water or probiotics. These results indicate that the dysbacteriosis mice could be established by ASC antibiotics treatment, while probiotics application has potent effect for rescuing the flora distribution.

**Table 1:** Primer sequences used in qPCR for bacterial identification

Target species	Sequence (5′–3′)
*Bifidobacterium*	
Forward	GCGTGCTTAACACATGCAAGTC
Reverse	CACCCGTTTCCAGGAGCTATT
*Escherichia coli*	
Forward	GGAGCAAACAGGATTAGATACCC
Reverse	AACCCAACATTTCACAACACG
*Lactobacillus*	
Forward	CGATGAGTGCTAGGTGTTGGA
Reverse	CAAGATGTCAAGACCTGGTAAG

### Dysbacteriosis induced mechanical allodynia and visceral pain

**Fig. 3:**
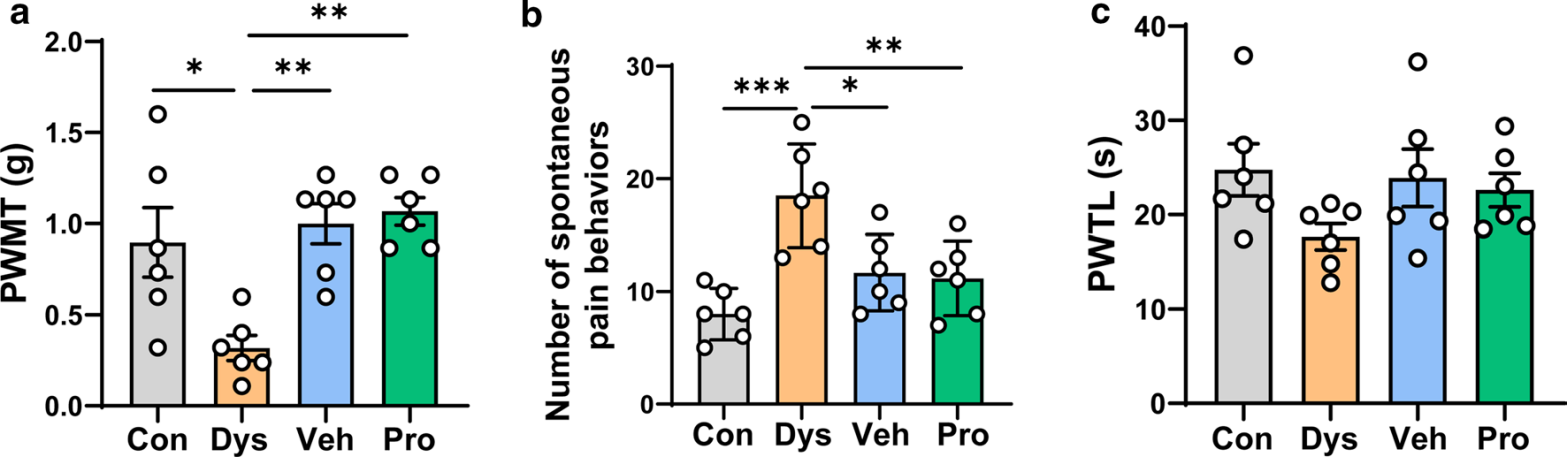
Vehicle and probiotics alleviated mechanical allodynia and visceral pain in dysbacteriosis mice. **a** The paw withdrawal mechanical threshold (PWMT) was reduced in Dys group but recovered in Veh and Pro group. **b** The number of spontaneous pain behaviors was increased in Dys group but recovered in Veh and Pro group. **c** No statistically significant difference of the paw withdrawal thermal latency (PWTL) was observed among the four groups. n = 6 mice mice per group (one-way ANOVA). *p < 0.05; **p < 0.01; ***p < 0.001

The possible mechanical allodynia, thermal hyperalgesia and visceral pain responses were then evaluated by measuring the paw withdrawal mechanical thresholds (PWMT) in *von frey* filament test, paw withdrawal thermal latency (PWTL) in hot plate test and spontaneous pain behavioral responses in open field test, respectively (Fig. 3). Visceral pain were defined by mouse postures including licking of the abdomen without other grooming behavior, flattening the abdomen against the floor, abdominal retractions, and whole-body stretching as described in the previous study[6]. The PWMT of Dys mice were significantly reduced as compared with Con mice, suggesting that dysbacteriosis induce a significant mechanical allodynia. While following treatment with 2-week drinking water or probiotics largely alleviated the allodynia (Fig. 3a). Similar results could be observed in the visceral pain test [12]. We found that ASC antibiotics-treated mice exhibited obvious visceral pain-related behaviors, which were alleviated by vehicle treatment or probiotics treatment (Fig. 3b). However, in hot plate test, the PWTLs were not different in mice in Con, Dys, Veh and Pro groups, although a decreased tendency of the PWTL was shown in Dys group in comparison with Con group (Fig. 3c). In total, these behavioral results show that obvious mechanical allodynia and spontaneous visceral pain are induced in dysbacteriosis mice with antibiotic application, which are rescued by following 2-week exposure to either drinking water or probiotics.

### Dysbacteriosis induced anxiety-like behavior

**Fig. 4:**
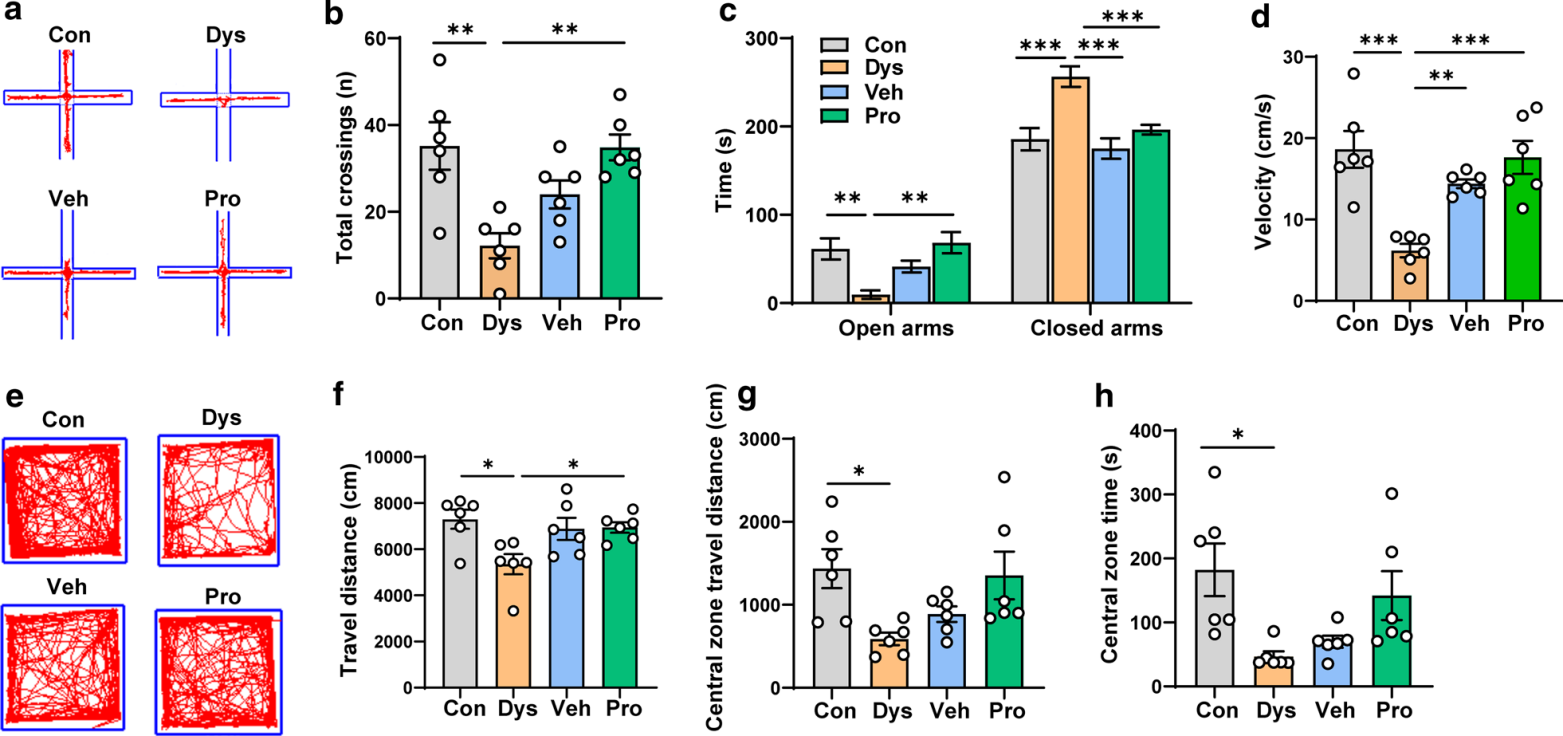
The enhanced anxiety-like behavior in dysbacteriosis mice. **a** Representative traces in the elevated plus maze test of different groups. The total crossings (**b**), time spent in open arms and closed arms (**c**), and velocity of mice (**d**) were summarized in different groups. **(E)** Representative traces in open field test of different groups. **f**–**h** The travel distance, central zone travel distance, and central zone time of mice were summarized in different group. n = 6 mice per group (one-way ANOVA for **b**, **d** and **f–h**, two-way ANOVA for **c**). *p < 0.05; **p < 0.01; ***p < 0.001

To test whether alternation of intestinal flora affect anxiety-like behavior in mice, we carried out elevated plus maze test and open field test [13]. In elevated plus maze (EPM) test, the total crossings and the time spent in open arms in Dys mice were lower than those of Con mice, while the time spent in closed arms in Dys mice was higher than that of Con mice (Fig. 4a–c). These results strongly suggest that a 3-week treatment of ASC antibiotics induced anxiety in Dys mice. Besides, following 2-week application of probiotics significantly reversed the anxiety behaviors, indicating the important role of probiotic treatment in alleviating anxiety induced by antibiotics in mice. Meanwhile, the velocity of Dys mice was significantly lower than that of mice in Con, Veh and Pro groups (Fig. 4d), indicating that Dysbacteriosis also reduce the locomotion of animals.

We further tested the locomotion behaviors in the open field test (Fig. 4e–h). Dys mice showed a significant decrease in total travel distance, central zone travel distance and central zone time, suggesting that anxiety is induced in mice exposed to ASC antibiotics. However, following exposure to vehicle or probiotics could only significantly increase the total travel distance but not the travel distance or time spent in central zone area, although an increased tendency for the central zone time and central zone distance could be easily noticed.

### Dysbacteriosis induced depression-like behavior

**Fig. 5:**
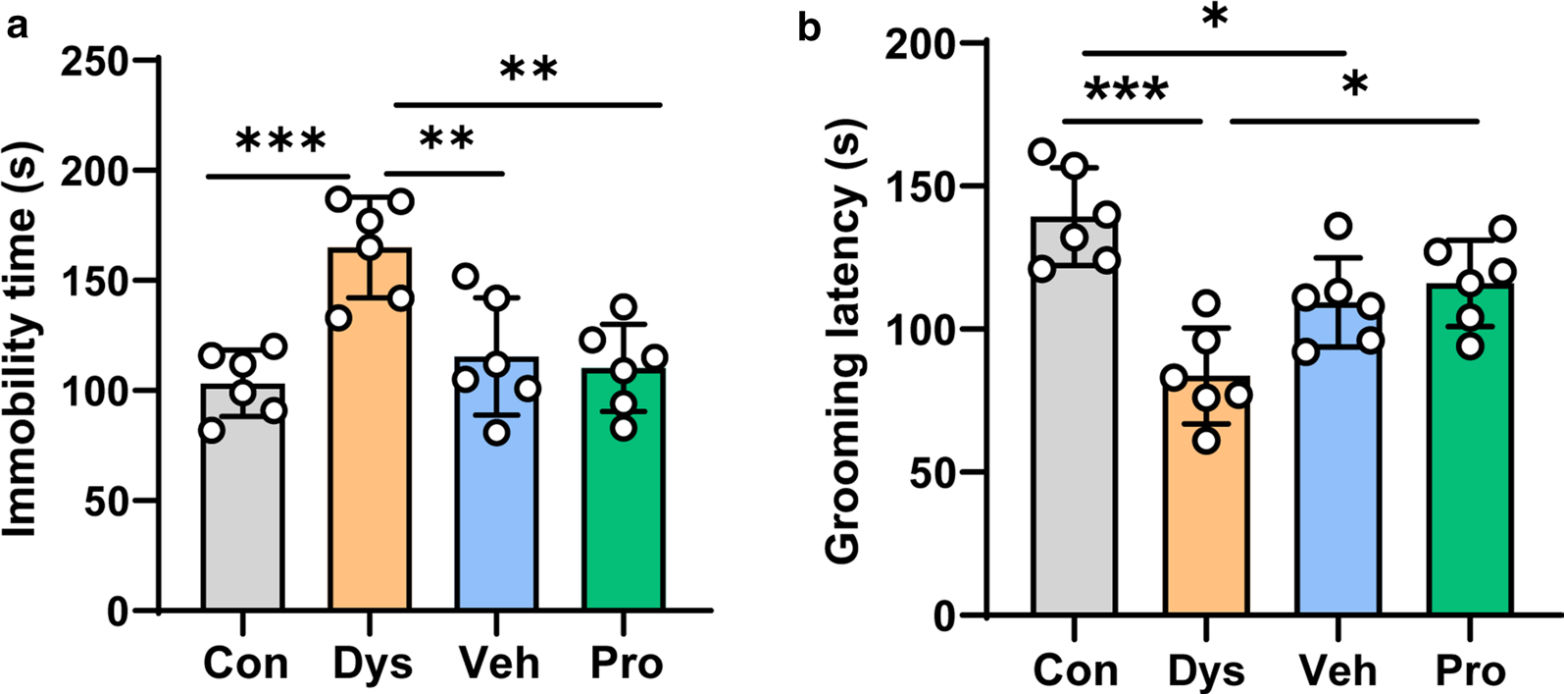
The enhanced depressive-like behavior in dysbacteriosis mice. **a** The length of absence of escape-oriented behavior in tail suspension test. **b** The grooming latency alternation of mice in different groups in splash test. n = 6 mice per group (one-way ANOVA). *p < 0.05; **p < 0.01; ***p < 0.001

Then, tail suspension test and splash test were performed to assess the depression-like behavior. The immobility time length of absence of escape-oriented behavior in tail suspension test and grooming latency in splash test were recorded [14]. As shown in Fig. 5, Dys mice exhibited increased immobility time (165.0 ± 9.349 s) and decreased grooming latency (83.67 ± 6.859 s), when comparing with mice in Con group (142.7 ± 5.493 s, 139.30 ± 6.955 s). These depression-like behaviors were largely rescued by following 2-week application of drinking water or probiotics.

### Dysbacteriosis damaged spatial memory performance

**Fig. 6:**
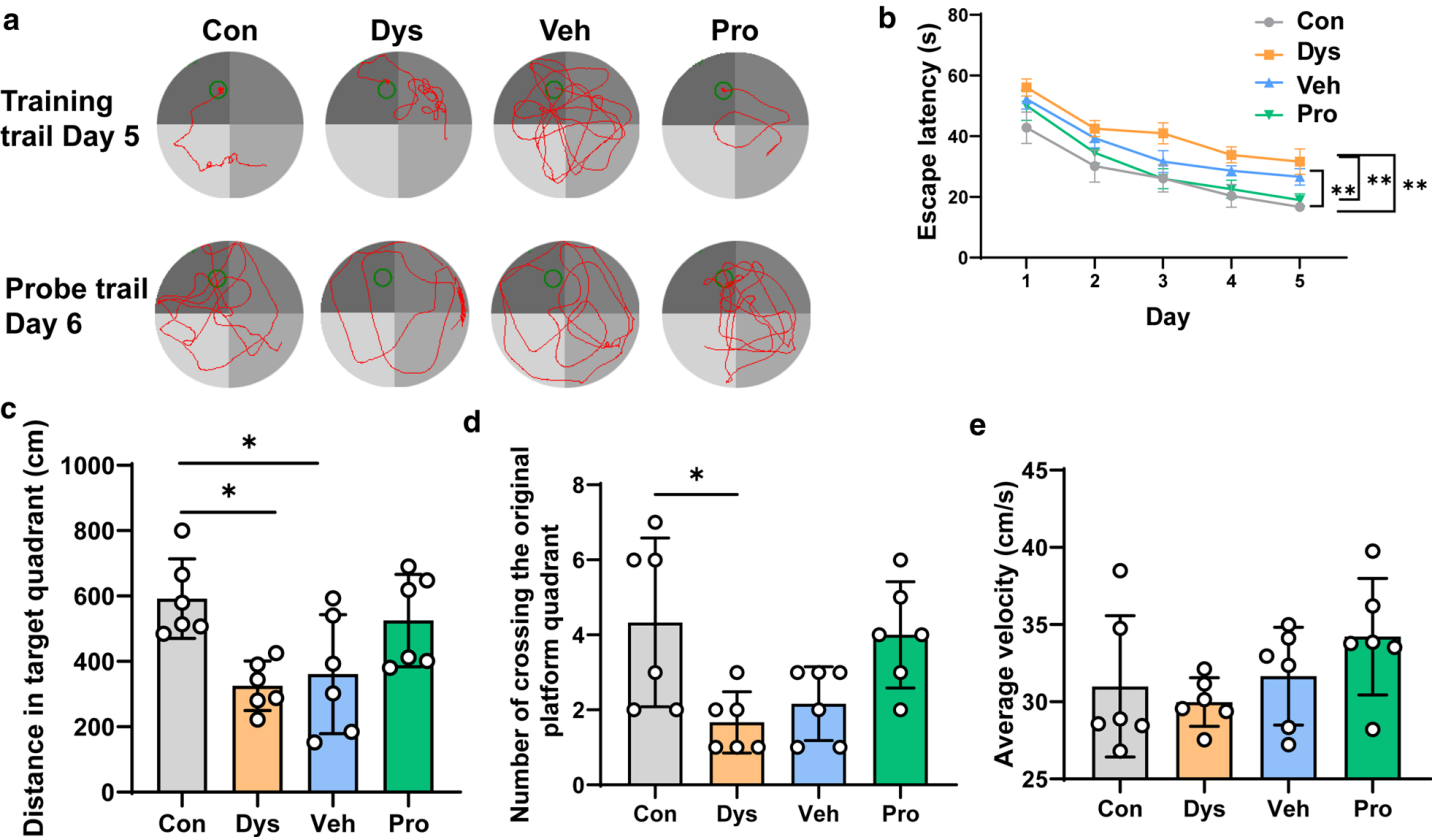
Effect of antibiotics and probiotics on spatial memory. **a** Representative travel paths of mice in different groups in training trail and probe trail. **b** Escape latency of training trail from Day 1 to Day 5. **b-d** Distance traveled in the target quadrant in probe trail on Day 6, Number of crossing the original platform quadrant in probe trail on Day 6, and Average velocity of mice in probe trail on Day 6 (**e**) were summarized in different groups. n = 6 mice per group (one-way ANOVA for **c–e**, two-way ANOVA for **b**). *p < 0.05; **p < 0.01

Next, hippocampus-dependent spatial memory was tested in Morris water maze [15]. The latency for identifying the location of a hidden platform with respect to surrounding contextual cues was calculated in the four groups over the 5-day training trail. It’s shown that the escape latency was increased in Dys mice when compared with mice in control group (Fig. 6a, b). During the probe trail, the distance traveled in target quadrant and number of crossing the original hidden platform quadrant were significantly decreased in intestinal dysbacteriosis mice, while the swim average velocity remained no statistically different between Dys mice and other groups (Fig. 6c–e). It was also noticed that the distance in target quadrant of Veh mice was reduced significantly when compared with mice in control group (Fig. 6c). Together, these results demonstrate that dysbacteriosis damage spatial memory performance, and it could be rescued by probiotic treatment but not plain water.

### Probiotic treatment modulates the expression of C-Fos protein in the central nervous system

**Fig. 7:**
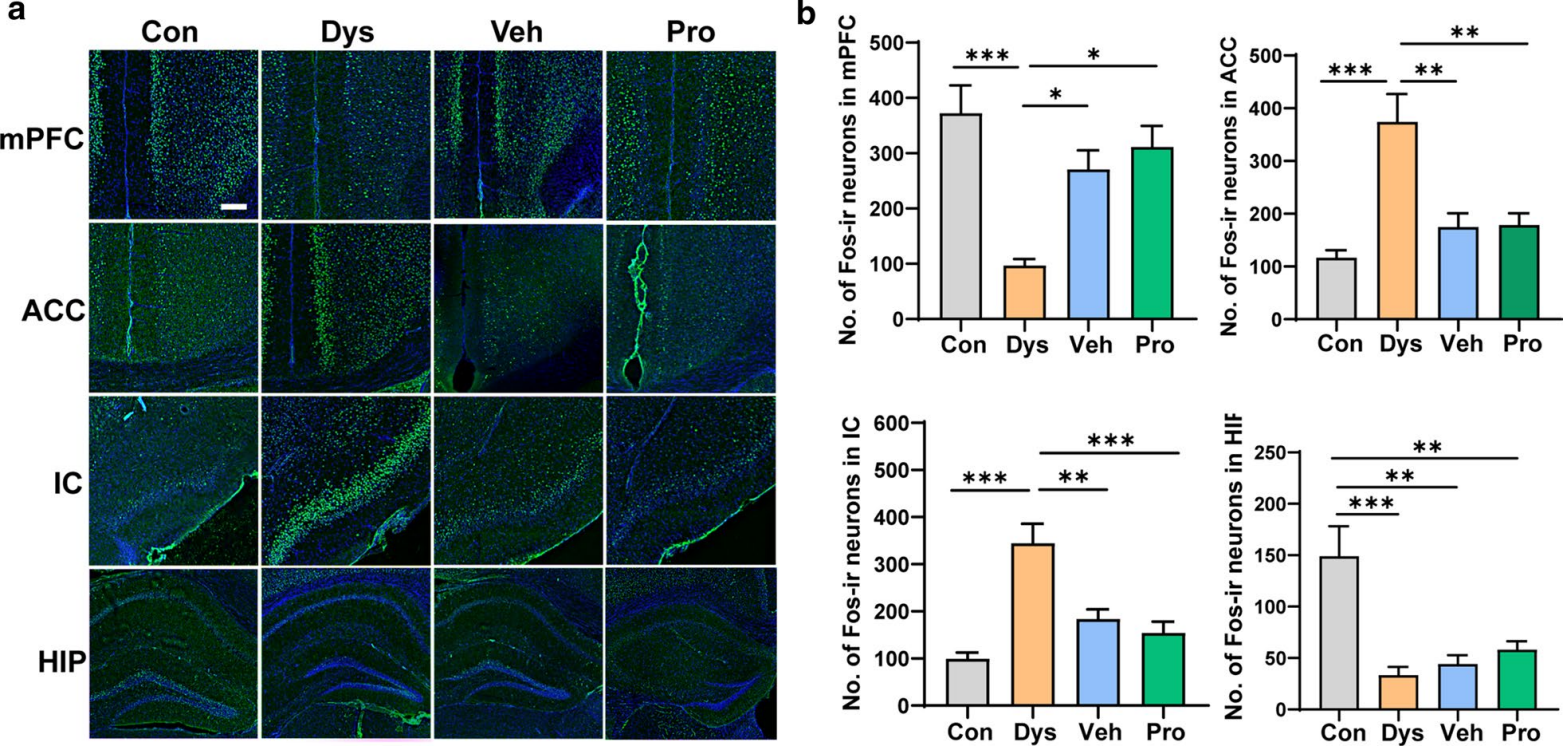
The expression of Fos protein was changed in different brain regions in antibiotics-treated mice. **a** Staining results of Fos in prefrontal cortex (PFC), anterior cingulate cortex (ACC), insular cortex (IC), and hippocampus (HIP). **b** Histogram showing the quantification of Fos-immunoreactive (Fos-ir) neurons in the above brain regions. n = 6 slices per mouse; 6 mice per group (one-way ANOVA). *p < 0.05; **p < 0.01; ***p < 0.001. Bar equals to 250 μm in **a**

Accumulating evidences show the involvement of cortical and subcortical areas, such as the anterior cingulate cortex (ACC), prefrontal cortex (PFC), and insular cortex (IC) in processing pain and emotional responces [16–18]. Substantial behavioral, electrophysiological, and imaging evidence supporting that the hippocampus (HIP) is implicated in several functions such as learning and memory, emotion and affect, and pain processing. We thus involved immunostaining for Fos protein, an activity-dependent neuronal marker [19], to explore the possibly changed brain areas involved with intestinal dysbacteriosis. We found that, the number of Fos immunoreactive (ir) neurons was significantly decreased in mPFC and HIP but increased in ACC and IC in Dys mice compared with the Con mice. The changed Fos expression pattern could be rescued by following application of Veh or Pro in mPFC, ACC and IC but not in the HIP (Fig. 7).

## Discussion

The composition of gut microbiome can be affected by numerous factors, such as changes in the diet, transplanting microbiota between animals, and exposure to antibiotics [20]. Among them, antibiotic treatment is the most commonly used method as it represents the simplest way to disturb gut microbial ecology [3]. Antibiotics are important medications to combat diseases caused by pathogenic bacteria, but they affect target pathogen and other beneficial species in the gut simultaneously [21]. Although probiotics have been proposed as a remedy for antibiotic-induced dysbacteriosis, the hazards of antibiotics and efficacy of probiotics remains uncertain. Most orally administered antibiotics alter the gut microbiota transiently during the treatment, and some antibiotics can induce long-lasting changes in the gut microbiota [22]. Thus, the results differs because of the mixture of antibiotics are not standardization and the measuring time points are different. In this study, a 3-week ASC antibiotics application was utilized to induce intestinal dysbacteriosis. In those Dys mice, enhanced anxiety-/depression-like behaviors and pain responses and damaged recognitive memory are shown, which are more or less recovered after withdrawal of antibiotics. Probiotics facilitates alleviation of anxiety, depression, mechanical allodynia and visceral spontaneous pain.

In our study, intestinal dysbacteriosis mice was established by ASC antibiotics application, which was assessed by the alternation of relative abundance of Bifidobacterium, Escherichia Coli, and Lactobacillus. The abundance of beneficial bacteria including Bifidobacterium and Lactobacillus decreased dramatically in Dys group and replenished in Pro group, which was in consistent with the previous study[8]. It has been reported that decreased Bifidobacterium and increased Escherichia could be observed in the fecal samples from patients with Alzheimer`s diseases and IBS patients [23, 24]. Bifidobacterium and Lactobacillus have been shown to decrease the visceromotor response to colorectal distention in animal models [25]. We now also showed that probiotics including Bifidobacterium and Lactobacillus could relieve visceral hypersensitivity and mechanical allodynia. Recent study demonstrates that Lactobacillus treatment alleviates anxiety and depressive behaviors [13], which correspond to the results of EPM, TST and splash test in the present study. As numerous studies showed that antibiotic application does not interfere with body weight significantly [9, 21, 26, 27]. Thus, we focused on the correlations of gut microbiota and behavioural changes and neglect the influence of antibiotics on body weight. Besides, as shown in Fig. 6a, b, the longer escape latency of the Dys mice in the training trail is not because of the lower locomotion, but more likely related to damaged learning and memory ability which can be figured out by the disordered traces compared with the Con mice.

It is necessary to investigate the relationship of behavioral alternations especially pain perception with antibiotics-induced neuronal activation in different brain regions, thus we conducted Fos mapping throughout the whole brain. In the present study, the changed Fos expression in the mentioned brain areas are in highly coincident with the behavioral performances. In detail, the decreased number of Fos-ir neurons in mPFC, reduced pain threshold and increased anxiety-like behaviors can be explained by the results that mPFC initiates the alleviation of chronic pain and anxiety-like behaviors[18]. Abundant literatures document that ACC and IC are crucial cortical areas for pain perception, deterioration, and its related emotional disorders such as aversive learning and fear [17, 28–30]. The results of this study confirmed that the activated ACC and IC are closely related to the intestinal dysbacteriosis induced pain and its associated anxiety. As shown in Figs. 6, 7, the hippocampal Fos expressions of Pro mice were not tightly correlate to the working memory performance as the escape latency of Pro mice were improved but the number of Fos-ir neurons stayed unchanged. We think that, although the Fos expression is an important indicator for showing the changed activity of neurons in most of the brain areas, it’s possible that the Fos expression is not coincident with the behavioral responses in all cases. It has been shown that the expression of Fos in the hippocampus is not a direct predictor or explicit to spatial memory learning [31].

Several different findings were also found in the present study, in comparison with previous works. Firstly, a recent study indicates that antibiotics plays an analgesia role but in the mice with CCI neuropathic pain model [5]. We propose that the difference may come from different animal model: they apply antibiotics in neuropathic pain mice while we use the normal mice to induce intestinal dysbacteriosis. The other reason may lie in the different composition and delivery way of the antibiotics. They feed the mice once daily with gastric gavage (0.5 g/L Neomycin, 0.5 g/L Ampicillin, 0.5 g/L Metronidazole, and 0.5 g/L Vancomycin), while we deliver the mice with a mixture of 1 g/L Ampicillin, Streptomycin, Clindamycin supplied ad libitum. Secondly, one previous study shows that no cognitive behaviors changed in rats in Morris water maze test 8 weeks after Vancomycin gavage [9], while another reports that the spatial memory is damaged in mice 1 week after Ampicillin gavage [8]. We think that the antibiotics utilized and the detection time point may be important to find the difference. In our study, changing the antibiotics to plain water for 2 weeks can result in a partially rescued phase of the damaged spatial memory. We hypothesize that when the time point come to 8 weeks after the antibiotics changing to plain water, there would be no significant differences between the Con group and Dys group. This may also explain the conflicting conclusion of the previous studies to some extent.

In sum, gut microbiota is important for pain responses and pain-related disorders, as well as recognitive performance in dysbacteriosis mice, both of which are deterioration by antibiotics-induced gut microbiota alternations. We also confirm that these alternations are transient and there exist the neuroprotective effect of probiotics against intestinal dysbacteriosis. Furthermore, gut microbiota can produce neuroactive compounds such as neurotransmitters including noradrenaline, γ-aminobutyric acid (GABA), dopamine and serotonin [32]. Thus, it is possible that the changed neurotransmitter release are involved in various neurological disorders caused by antibiotics treatment, which would be further explored in our future study.

## Materials and methods

### Animals

Male C57BL/6J (6 weeks old) mice were purchased from the Experimental Animal Center of Air Force Medical University (Xi`an, China). They were housed in plastic cages under controlled laboratory conditions (temperature: 21–24℃; 12-h light/dark cycle: lights on 9 a.m.-9 p.m.) with food and water supplied ad libitum. After one-week acclimatization, mice were randomly divided into four groups with nine mice each, namely Group A (control group; plain water for 3-week), group B (dysbacteriosis group; water with ASC antibiotics for 3-week), Group C (saline group; water with ASC antibiotics for 3-week and then plain water for 2-week), Group D (probiotics group; water with ASC antibiotics for 3-week and then probiotics for 2-week). The ASC antimicrobial cocktail is a mixture of Ampicillin (Macklin, Shanghai, China), Streptomycin (Macklin, Shanghai, China), Clindamycin (Macklin, Shanghai, China), and sterile drinking water at a final concentration of 1 g/L as previously described[4, 11]. While in Group C and D, there was a reverse course with ASC antibiotic mixture changed to drinking water or probiotics (Live Combined Bifidobacterium and Lactobacillus Tablets, Inner Mongolia Shuang Qi Pharmaceutical Co., Ltd. PRC, Inner Mongolia, China) for another 2 weeks. The mice in each group were then subjected to gut microbiota analysis, a series of behavioral tests including anxiety, depression, pain response test and recognitive memory test and immunostaining for Fos protein.

To minimize environment stress, all mice in each group (N = 6 mice) were permitted to adapt to the environment of behavioral test room (12 h light/dark cycle, 22℃, 50% relative air humidity) 3 days before testing. Mice were subjected to a series of behavioral tests during the following weeks as shown in Fig. 1.

### Anxiety-like behavior

Elevated plus maze (EPM). The following day, the mice were subjected to EPM test system consisted of two open arms (30 cm × 5 cm) and two closed arms (30 cm × 5 cm × 15 cm) as described in previous studies [33]. They were individually placed in the central square platform (5 cm × 5 cm) 70 cm above the floor with the nose toward a closed arm and allowed to explore for 5 min. The time spent and total distance traveled were recorded using a motion tracking system and calculated by the analyzing system (Shanghai Mobile Datum Information Technology, Shanghai, China).

Open field test (OFT). The mice were placed in the OFT system, which was comprised of 8 square chambers (50 cm × 50 cm × 45 cm) [6]. Their horizontal movement was detected by a motion tracking system and the central distance and total traveling distance were analyzed by the analysis software (Shanghai Mobile Datum Information Technology, Shanghai, China).

### Depression-like behavior

Tail suspension test (TST). Mice were suspended 50 cm above the floor using adhesive tape placed approximately 1 cm from the tip of the tail for 5 min. The duration of immobility was monitored and recorded in seconds by a time recorder. Immobility of the mice was defined as the absence of escape-oriented movement and completely motionless while suspended.

Splash Test. This test was based on grooming behavior, which was performed by vaporization of the 10% of sucrose solution on the dorsal coat of mice as described in previous study[14]. The latency and frequency of the grooming behavior was scored during 5 min and then analyzed.

### Recognitive memory

Morris water maze test (MWMT). Learning and memory performance was evaluated in a circular tank (diameter, 120 cm) filled with white opaque water at approximately 21℃. A fixed platform (diameter, 10 cm) remained constant and submerged 1 cm below the water surface in a target quadrant. Reference cues of different colors and shapes were placed along the walls surrounding the tank. During the hidden platform testing, mice were gently placed into the tank and the entry point of four quadrants were randomly changed. Mice were gently guided to the hidden platform if they failed to find it within 60 s, and they were kept on the platform for 15 s. During training trails, the mouse behavior was recorded using a motion tracking system and the escape latency was calculated by the analyzing system (Shanghai Mobile Datum Information Technology, Shanghai, China) per testing day. On day 6, the hidden platform was removed and a probe test was performed. The swimming velocity and the number of crossing the area where the hidden platform used to be was recorded.

### Pain behavior

Paw withdrawal thresholds test. The mice were kept in Lucite cubicles over a wire mesh and acclimated for 30 min as described in previous studies[28, 34]. A series of *Von Frey* filaments (0.008, 0.02, 0.04, 0.16, 0.4, 0.6, 1, 1.4, 2 g) with various bending forces (according to 0.078, 0.196, 0.392, 1.568, 3.92, 5.88, 9.8, 13.72, 19.6 mN) were applied to the plantar surface of the hindpaw until the mice withdrew from the stimulus. The lowest force at which a withdrawal response was obtained was considered as the pas withdrawal threshold.

### Spontaneous pain

Visceral pain were defined by mouse postures including licking of the abdomen without other grooming behavior, flattening the abdomen against the floor, abdominal retractions, and whole-body stretching as described in the previous study[6].

### 16S rRNA qPCR

The time points of fecal samples collected of the four groups were shown in Fig. 1. DNA was extracted from stool samples that snap-frozened on dry ice using the TIANamp Stool DNA kit (Tiangen Biotechnology company, Beijing, China) according to the manufacturer`s instructions. The abundance of specific bacterial groups was measured by qPCR using genus-specific 16S rRNA gene primers (Table 1) and the SuperReal PreMix Plus SYBR Green kit (Tiangen Biotechnology company, Beijing, China). qPCR was performed in a real-time PCR detection system (Bio-Rad, Hercules, CA). Bacterial DNA was quantified using standard curves constructed with reference bacteria specific for each bacterial group analyzed.

### Fos mapping

Histology. Mice of the three groups were deeply anesthetized and then transcardially perfused with 0.9% saline (50 ml), followed by 4% paraformaldehyde (PFA, 100 ml, PH 7.4). Brains were extracted immediately, post-fixed in 4% PFA for 4 h, and cryoprotected by 0.1 M PB containing 4% (w/v) sucrose at 4℃ until cutting. 30 μm serial coronal sections were cut using a freezing microtome (Kryostat 1720, Leitz, Mannheim, Germany). Subsequently, the sections were washed with 0.01 M phosphate buffer solution (PBS, PH 7.4) and rinsed three times for 10 min. The sections were pre-incubated in blocking solution (PBS containing 1% bovine serum albumin, 0.3% Triton X-100) for 1 h, and then incubated with primary anti-c-Fos antiserum (1:400, Abcam, Cambridge, MA, USA) overnight. Next, the sections were rinsed and then incubated in an Alexa Fluor (AF) 488-conjugated Rabbit anti-mouse secondary antibody solutions (1:400) for 4 h at room temperature. The slices were then washed extensively with PBS, incubated with DAPI (D9542, Sigma, USA) at 1:1000 in PBS, and then mounted on microscope slides and cover-slipped. Images were acquired by using a virtual slide microscope (VS120, Olympus, Japan). The images were analyzed by using software CellSens Dimension (Olympus, Japan).

### Statistical analysis

All data were collected by experimenters who were blind to the conditions of the experimental animals. Statistical analysis was performed using GraphPad Prism 8 (version 8.0.2). Statistical significance was assessed by one-way ANOVA (Tukey`s multiple comparisons test) and two-way ANOVA (Sidak`s multiple comparisons test). Analyzed numbers (n) for each experiment are illustrated in the main text sections of corresponding figure legends. Data were presented as the Mean ± S.E.M. A threshold for statistical significance of *P* < 0.05 was chosen in this study.

## Data Availability

The data that support the findings of this study are available from the corresponding author upon reasonable request.
