# Correction to: Antibiotics-induced intestinal dysbacteriosis caused behavioral alternations and neuronal activation in different brain regions in mice

**DOI:** 10.1186/s13041-021-00781-y

**Published:** 2021-04-13

**Authors:** Pan Wang, Ke Tu, Peng Cao, Yuefan Yang, Hao Zhang, Xin-Tong Qiu, Ming-Ming Zhang, Xiao-Jun Wu, Hui Yang, Tao Chen

**Affiliations:** 1 Institute of Medical Research, Northwestern Polytechnical University, Xi`an, 710072 Shaanxi People’s Republic of China; 2 Department of Human Anatomy, Histology and Embryology & K.K. Leung Brain Research Centre, The Air Force Medical University, No. 169 Changle West Road, Xi’an, 710032 China; 3 Department of Anesthesiology, General Hospital of Tibet Military District, Lhasa, 850007 Tibet People’s Republic of China; 4 Department of Biomedical Engineering, The Air Force Medical University, Xi`an, China; 5 Key Laboratory for Space Bioscience and Biotechnology, School of Life Sciences, Northwestern Polytechnical University, Xi`an, China; 6 Department of Neurosurgery, Fudan University Shanghai Cancer Center, 270 Dongan Road, Xuhui, Shanghai, 200032 China

## Correction to: Mol Brain (2021) 14:49 https://doi.org/10.1186/s13041-021-00759-w

Following publication of the original article [1], it was flagged that due to a typesetting mistake, the equal contributions in the author group were omitted from the PDF version of the article: Pan Wang, Ke Tu and Peng Cao should have been listed as equal contributors.

The author group has been updated above and the original article [1] has been corrected. The publisher apologises to the authors and readers for the inconvenience caused by this omission.

## References

[1] Wang P (2021). Antibiotics-induced intestinal dysbacteriosis caused behavioral alternations and neuronal activation in different brain regions in mice. Mol Brain.

